# Comparison of discriminatory power and accuracy of three lung cancer risk models

**DOI:** 10.1038/sj.bjc.6605759

**Published:** 2010-06-29

**Authors:** A M D'Amelio, A Cassidy, K Asomaning, O Y Raji, S W Duffy, J K Field, M R Spitz, D Christiani, C J Etzel

**Affiliations:** 1 Department of Epidemiology, UT MD Anderson Cancer Center, 1155 Pressler Street – Unit 1340, Houston, Texas 77030-4009, USA; 2 Roy Castle Lung Cancer Research Programme, School of Cancer Studies, The University of Liverpool Cancer Research Centre, Liverpool L3 9TA, UK; 3 Departments of Environmental Health and Epidemiology, Harvard School of Public Health, Boston, Massachusetts 02115, USA; 4 Cancer Research UK Centre for EMS, Queen Mary University of London, Wolfson Institute of Preventive Medicine, Barts and the London School of Medicine and Dentistry, Charterhouse Square, London EC1 M 6BQ, UK

**Keywords:** lung cancer, risk model, 5-year lung cancer risk, relative risks, discriminatory power

## Abstract

**Background:**

Three lung cancer (LC) models have recently been constructed to predict an individual's absolute risk of LC within a defined period. Given their potential application in prevention strategies, a comparison of their accuracy in an independent population is important.

**Methods:**

We used data for 3197 patients with LC and 1703 cancer-free controls recruited to an ongoing case–control study at the Harvard School of Public Health and Massachusetts General Hospital. We estimated the 5-year LC risk for each risk model and compared the discriminatory power, accuracy, and clinical utility of these models.

**Results:**

Overall, the Liverpool Lung Project (LLP) and Spitz models had comparable discriminatory power (0.69), whereas the Bach model had significantly lower power (0.66; *P*=0.02). Positive predictive values were highest with the Spitz models, whereas negative predictive values were highest with the LLP model. The Spitz and Bach models had lower sensitivity but better specificity than did the LLP model.

**Conclusion:**

We observed modest differences in discriminatory power among the three LC risk models, but discriminatory powers were moderate at best, highlighting the difficulty in developing effective risk models.

Worldwide, an estimated 1.35 million new lung cancer (LC) cases and 1.18 million LC-related deaths occur every year (Parkin *et al*, 2005). It has been suggested that ∼70% of all LCs could be prevented by reducing the prevalence of major risk factors, particularly smoking (Danaei *et al*, 2005). Given that LC risk differs greatly among smokers, the ability to estimate an individual's absolute risk could be used to guide preventive interventions. In particular, absolute risk scores could be used both to motivate individuals to reduce their LC risk through behaviour and lifestyle modifications and to refine selection of participants for LC screening trials on the basis of maximising benefit (Vickers *et al*, 2006; Duffy *et al*, 2009). Other cancers that have well-known risk-prediction models include breast (Gail *et al*, 1989; Tyrer *et al*, 2004; Tice *et al*, 2005), colorectal (Imperiale *et al*, 2000; Selvachandran *et al*, 2002), melanoma (Cho *et al*, 2005; Fears *et al*, 2006), ovarian (Hartge *et al*, 1994), and bladder cancers (Wu *et al*, 2007).

Within the last decade, three models to estimate an individual's absolute LC risk were developed: the Bach (Bach *et al*, 2003), Spitz (Spitz *et al*, 2007), and the Liverpool Lung Project (LLP) models (Cassidy *et al*, 2008). All the three models share risk factors (such as smoking duration and occupational exposure to asbestos); however, differences arise, with the inclusion of lung-related comorbidities or family history information. These models have not previously been compared in an independent data set in terms of discriminatory power, accuracy, and clinical utility. Such a comparison, to evaluate whether these published risk models have similar discriminatory power for a given population of individuals, is important, given the potential application of risk-prediction models to strategies for primary and secondary preventions.

In this study, we used each of these models to estimate 5-year absolute LC risks for an independent population of LC patients and healthy controls. We compared the discriminatory power of these three models by calculating the area under the curve (AUC) of the receiver operator characteristic (ROC) curve for each 5-year absolute risk estimate. We evaluated the accuracy and compared the positive predictive value (PPV; the probability of accurately categorising an affected participant) and the negative predictive value (NPV; the probability of accurately categorising an unaffected participant) among the three risk models; we also evaluated the clinical utility of each.

## Materials and methods

A total of 4900 LC patients and controls were accrued for this study, 3197 were treated in the Thoracic Surgery, Thoracic Oncology, or Pulmonary Units at the Massachusetts General Hospital (MGH) (Boston, MA, USA). Starting in 1992, enrolment was initially restricted to patients with operable LC; however, case definition was expanded in August 1996 to include inoperable LC to reflect the full spectrum of LC patients. Lung cancer diagnosis was histologically confirmed by a lung pathologist. Controls (*N*=1703) were LC-free individuals initially accrued from among family members or friends of cases, but accrual was subsequently expanded to include friends and family (not blood related to study cases) of individuals being treated at the MGH for non-LC diseases (Xu *et al*, 1996; Garcia-Closas *et al*, 1997; Wang *et al*, 2001).

Inclusion of risk factors in the three LC risk models is summarised in Table 1. Smokers were defined as those who had smoked >400 cigarettes in their lifetime; former smokers were those who had quit smoking at least 1 year before the cancer diagnosis (patients) or the interview (controls). Smoking duration was determined by subtracting the age at which the participant had started smoking from either the age at which the participant had quit smoking (former smokers) or the participant's current age (current smokers). Pack-years were calculated by multiplying the smoking duration (in years) by the number of cigarettes smoked per day and then dividing by 20. Time of smoking cessation for former smokers was determined by subtracting the age at which the participant had quit smoking from the participant's current age.

Participants were classified as positive for asbestos exposure if they had been directly exposed for at least 8 h per week for a year or if they were employed in an asbestos-related industry (according to the Standard Industrial Classification Manual (1972) and/or the Dictionary of Occupational Titles (1991)). Exposure to wood dusts (including sawdust or sanding dust) for at least 8 h per week for a year was self-reported, or for a family history of any cancer if at least two first-degree relatives had cancer. Participants were classified as positive for a family history of any smoking-related cancer if at least one first-degree relative had had cancer at some point in his or her life. Participants were also classified by self-reported physician-diagnosed emphysema or hay fever at any time before study entry (Spitz) or by physician-diagnosed pneumonia at least 2 years before entry (LLP).

Any study participant with missing data for any of the risk factors for any model was excluded from analysis. As all three models were developed using data obtained from White participants, we only included individuals who self-reported being non-Hispanic White. For the comparison of discriminatory power between the LLP and Bach models and the Spitz and Bach models only ever smokers (total of 1066 LC and 677 controls) were used as the Bach model was developed only for ever smokers (Bach *et al*, 2003). In the comparison between the LLP and Spitz models, never, former, and current smokers were included (total of 1121 LC and 1024 controls).

The institutional review boards at the M. D. Anderson Cancer Center, MGH, and the Harvard School of Public Health approved this study.

We calculated the 5-year absolute risk of LC for the three models, using MatLab software (The MathWorks Inc., Natick, MA, USA). For the Bach model, they were obtained by running 1-year incidence and mortality models recursively five times, with each individual contributing to the predicted risk for 5 years. For the Spitz and LLP models, they were estimated by combining the risk of cancer from the relative risk model with age- and gender-specific LC incidence rates.

Details regarding the exact calculation of risk for each model were given in the original paper (Bach *et al*, 2003; Spitz *et al*, 2007; Cassidy *et al*, 2008). With the LLP model, the *α*-value used to calculate the 5-year absolute risk was adjusted for the US LC incidence rate (Appendix Table A1). For each participant, we had three estimates of absolute risk for LC (one from each model). For each model, we used NCSS statistical software (NCSS, Kaysville, UT, USA) to calculate the specificity and sensitivity required to construct ROC curves and estimate AUC (binomial method) and the 95% confidence interval (95% CI) for each of the three models. We also calculated the AUC after stratification of participants by sex and age (<50 *vs* ⩾50 years). We then conducted pairwise comparisons of the AUCs of the three models using the method described in the NCSS package (Hanley and McNeil, 1983), the test statistic for comparing two ROC curves being given by 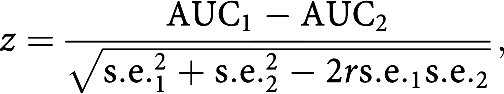 where AUC_*i*_ is the area under the ROC curve from the *i*th model (*i*=1,2), s.e._*i*_ the s.e. of AUC_*i*_, and *r* the correlation between AUC_1_ and AUC_2_ (Tyrer *et al*, 2004). The test statistic *z* follows a standard normal distribution; and values of *z*>z_1−*α*_ are interpreted as evidence that AUC_1_ is significantly greater than AUC_2_ for the given *α*-level.

From these risk factors, a relative risk was calculated and combined with age- and gender-specific incidence rates from x (SEER) (SEER, 2005), and all-cause mortality (excluding LC) rates from CDC (Centers for Disease Control) to estimate the absolute risk of LC (National Center for Health Statistics, 2003) (Appendix Table A2). As most of the absolute risk calculations involve pairwise comparisons from three different LC models, the Bonferroni correction was taken into account to adjust for any multiple comparisons issues.

We calculated the PPV and NPV for each of the models (Spitz, Bach, and LLP) separately for all participants and then stratified them by smoking status (former and current). We conducted pairwise comparisons (Spitz *vs* LLP, Spitz *vs* Bach, and Bach *vs* LLP) of the PPV and NPV to test differences of these two statistics among the three models using the normal approximation to the test of two proportions. As with the absolute risk results, the Bonferroni correction was used for both the PPV and the NPV to adjust for multiple testing. Clinical utility of the models was evaluated using scaled rectangle diagrams as implemented in the Search Partition Analysis (SPAN, Auckland, New Zealand) program (Marshall, 2001, 2005, 2009). Scaled rectangle diagrams display the joint occurrence of attributes (namely risk for disease) for a risk model and true disease status and provide a visual presentation of how well a model discriminates. With these diagrams, the white rectangle represents all individuals, the green rectangle represents all cases, and the blue, purple, and red rectangles represent individuals with three increasing levels of LC risk (2.5, 5, and 7.5%, respectively). Models with high clinical utility will have the vast majority of their cases, have higher levels of risk, and have fewer controls with those individuals at the higher LC absolute risk.

## Results

The epidemiological and demographic data for the validation set of 1066 LC patients and 677 controls are presented in Table 2. Patients (mean age, 64.8 years) were older than controls (mean age, 61.1 years; *P*<0.001). The majority of patients (58%) and controls (52%) were male. There was a higher percentage of former smokers among controls (74.2%) than among patients (56.2% *P*<0.001). Lung cancer patients who were current smokers smoked significantly more cigarettes per day (mean, 29.9), and had smoked for longer periods (mean, 43.8 years) than did controls (mean cigarettes smoked per day: 21.1, *P*<0.001; smoking duration: 38.5 years, *P*<0.001). Similarly, patients who were former smokers had smoked significantly more cigarettes per day (mean, 30.6) and had smoked for longer periods (mean, 34.8 years) than did controls (mean cigarettes smoked per day: 22.9, *P*<0.001; smoking duration: 24.2 years, *P*<0.001). Lung cancer patient pack-years were over 24 units higher in both current and former smokers than in controls, and these differences were highly significant in both smoking groups (*P*<0.001). Controls reported longer quitting durations (mean, 19.8 years) than did patients (mean, 14.1 years; *P*<0.001). Former smokers more after reported a family history of any cancer (34.4%) and smoking-related cancers (30.6%) than did controls (any cancer: 27.9%, *P*=0.023; smoking-related cancers: 22.9%, *P*=0.005); and current smokers reported a significantly higher percentage of smoking-related cancers (30.4%) than did controls (22.3%, *P*=0.049).

The discriminatory power for the three models, overall and stratified by smoking, age, and sex, are summarised in Table 3, the AUCs being 0.69 for the Spitz (95% CI=0.66–0.71) and LLP (95% CI=0.67–0.71) models and 0.66 (95% CI=0.64–0.69) for the Bach model. The differences in discriminatory power between the LLP and Bach models were significant (*P*=0.023), and the differences between the Spitz and Bach models reached borderline significance (*P*=0.072). Among former smokers, the discriminatory power was 0.70 (95% CI=0.67–0.73) for the Spitz and LLP models and 0.65 (95% CI=0.62–0.68) for the Bach model. Among current smokers, the discriminatory power was 0.68 (95% CI=0.64–0.72) for the Spitz model, 0.65 (95% CI=0.60–0.69) for the Bach model, and 0.66 (95% CI=0.62–0.70) for the LLP model. Among former smokers, the Bach model was outperformed by both the LLP (*P*=0.002) and the Spitz (*P*=0.008) models, whereas among current smokers, only the Spitz model significantly outperformed the Bach model (*P*=0.024). When incorporating never smokers for testing discriminatory power, the LLP model (AUC=0.72, 95% CI=0.70–0.74) outperformed the Spitz model (AUC=0.68, 95% CI=0.66–0.71) significantly (*P*=0.001).

We also tested the discriminatory power of all models when participants were stratified by age and sex (Table 3) and for women over the age of 50 years, observed significant differences in discriminatory power between the Spitz and Bach models, and the LLP and Bach models.

Table 4 summarises the NPV and PPV results for each. Overall, the three models had reasonable PPV levels (all >70%); the Spitz model had a significantly higher PPV (88.2%) than those of the LLP (75.9% *P*<0.001) and the Bach (80.9% *P*=0.009) models. Among former smokers, the Spitz model had significantly higher PPV (85.5%) than did the LLP model (72.6% *P*<0.001) but not significantly higher PPV than the Bach model (83.6% *P*=0.851). However, among current smokers, the Spitz model had higher PPV (91.9%) than did the Bach (80.4% *P*=0.002) and the LLP (80.9% *P*<0.001) models. The overall NPV for each of the three models were lower than the PPV (range=45.0–56.0%), with the LLP model having a substantially better probability of accurately categorising an unaffected participant. The LLP model was also significantly better for the NPV among former smokers, but both the Spitz and Bach models were competitive with the LLP model in calculating the NPV among current smokers.

**Figure 1:**
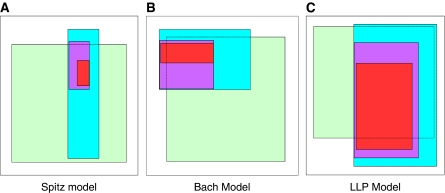
Clinical utility of the Spitz, Bach, and LLP models. Scaled rectangle diagrams for (**A**) the Spitz, (**B**) Bach, and (**C**) LLP risk models at defined levels of lung cancer risk. For each colour of the diagram: white equals all controls with <2.5% risk, and green equals all cases with <2.5% risk. Blue represents all individuals with at least 2.5% risk, but <5% risk. Purple represents all individuals with at least 5.0% risk, but <7.5% risk. Red represents all individuals with at least 7.5% risk.

To demonstrate the clinical utility of each model, Table 5 presents the percentages of patients and controls with LC risk estimates of >2.5, 5, and 7.5% as determined by each model. Using a cutoff of >2.5% risk as an example, the percentages of LC patients that were correctly identified by the Spitz, Bach, and LLP risk models were 26.6, 30.2, and 66.7%, respectively. The percentages of controls with >2.5% risk that were incorrectly identified as LC patients by the Spitz, Bach, and LLP risk models were 5.6, 11.2, and 33.4%, respectively. For all three models, setting a higher risk cutoff resulted in a lower proportion of controls being incorrectly identified as LC patients and a lower proportion of LC patients being correctly identified. This is evident in the scaled rectangle diagrams for the Spitz, Bach, and LLP risk models at cutoffs of >2.5, 5, and 7.5% absolute risk, respectively (Figure 1). Using the >2.5% risk cutoff, the LLP model identified 276 LC patients who were not identified by the Spitz and Bach models, but it also incorrectly identified 139 controls as LC patients. Although the Spitz and Bach models identified fewer LC patients (17 and 15, respectively), significantly fewer controls were incorrectly identified as patients (5 and 8, respectively) compared with the LLP model. Using the >7.5% risk cutoff, the Spitz model had 100% specificity, but its sensitivity was impractically low (2.2%). At this level of risk, for every four LC patients correctly identified by the LLP model, one control was incorrectly identified as a LC patient, wherein as the equivalent patient-to-control ratio for the Bach model was 5 to 1.

## Discussion

The purpose of this analysis was to compare the discriminatory power and accuracy of three LC risk-prediction models using an external set of LC cases and controls. We observed that the Spitz and LLP models had similar abilities to discriminate between former and current smoking cases and controls and that each of these models outperformed the Bach model. The Spitz and LLP models incorporated population-based incident LC rates, which could account for their better discriminatory power than that of the Bach model. For every 5-year age group from 20 to 89 years, we incorporated the SEER rates for the incidence of LC and the mortality rates from all causes other than LC (Appendix Table A1). In terms of model accuracy, the Spitz model had higher PPV than did the LLP and Bach models among both types of ever smokers, but the LLP model outperformed both the Spitz and Bach models in terms of the NPV. In terms of clinical utility, the Spitz model had the lowest false-positive rate for risk estimates >2.5%, whereas the LLP model had the highest false-positive rate. At all levels of risk, the LLP model correctly identified a higher proportion of LC patients than did the other models did but also incorrectly identified a higher proportion of controls as LC patients.

Each model included some form of tobacco exposure. In the Bach model, the variables – duration of smoking (in years) and number of cigarettes smoked per day – are included for both former and current smokers. In the Spitz model (controls matched to cases on smoking status), the duration of smoking and numbers of cigarettes smoked per day are combined into pack-years for current smokers only and into age at smoking cessation for former smokers. The Bach and LLP models do not include a smoking cessation variable. The Bach model included smokers aged 50–75 years who are/were heavy smokers (10–60 cigarettes per day for 25–60 years) and who had quit no more than 20 years previously (Bach *et al*, 2003).

In terms of clinical utility, the Spitz and Bach models performed reasonably well in identifying LC patients at defined levels of risk while limiting the number of false-positive results. However, the LLP model was much better at identifying individuals with LC but also had a much higher false-positive rate than the Spitz and Bach models had. This could be attributed to the importance of smoking in the LLP model. The Spitz model's relatively low recognition of cancer patients with a >2.5% absolute LC risk could be caused by smoking being a matching variable in the model rather than a risk factor. The overall high (>75%) PPVs for the three models indicate that they can identify high-risk individuals; however, the overall relatively low NPVs (between 45 and 56%) indicate that many low-risk individuals would be identified as well. The scaled rectangle diagrams illustrate more clearly the modest discriminatory performances of the Spitz, Bach, and LLP models and provide a sobering message about LC risk prediction. To substantially improve LC risk discriminatory power for individual patients, we need to identify a risk factor (other than smoking habits) that has a different distribution in LC patients from those who will not develop it; to date, there is no evidence for such a factor. High expectations have been pinned on genome-wide association studies, which have successfully identified hundreds of common genetic variants that are strongly associated with the risk of more than 40 diseases, including LC (Kraft and Hunter, 2009). However, a strong association does not necessarily guarantee good classification or discriminatory ability (Jakobsdottir *et al*, 2009). It was recently shown that on average, 80 common variants with odds ratios of 1.25 each were required to develop a model useful for the identification of high-risk individuals (AUC>0.80) for genetic profiling studies (Janssens *et al*, 2006).

Our study had some limitations. The most important limitation is that the study design is a case–control study, which could lead to some recall bias with the self-reported variables, such as smoking and environmental tobacco smoke exposure (Asomaning *et al*, 2008). However, in this study, controls were recruited from family and friends of those being treated for LC at the MGH, so that exposures for the self-reported variables would be similar or non-differential, among cases and controls, which would limit recall bias (Miller *et al*, 2003). With non-differential biases, AUC results will regress to the null, so it is possible that the AUC results are conservative instead of overstated (Greenland and Lash, 2008).

Other minor limitations include that the risk-prediction models compared in our study were developed in Caucasian populations, so the validation was also restricted to Caucasians, and thus, the models may not be applicable to other racial or ethnic groups. In addition, for most of the analysis, we only included ever-smoker cases and controls in our analysis, and thus, the enormous contribution of smoking to LC risk was effectively underestimated. The ultimate test of a model's application is its accurate prediction of risk in an independent data set. However, direct comparison of risk models is complicated by the fact that few studies have population samples that are large enough and diverse enough in age and risk factor backgrounds (Cassidy *et al*, 2007). Thus, to avoid possible information bias, it was imperative for our analysis to select only patients and controls from those who had complete information relating to risk-model covariates.

Despite these limitations, our analyses showed that LC risk-prediction models performed reasonably well when compared with each other in an independent validation set. All models include biologically plausible and well-established risk factors that have been shown to be significant in previous studies. One possible caveat is that the discriminatory values do not exceed 0.75, a value that has been suggested for the screening of individuals with an increased risk of disease (Janssens *et al*, 2007). This relatively low discriminatory value suggests that there is much work yet to be accomplished in LC risk prediction, especially compared with other cancer risk models such as colorectal cancer, which has a concordance statistic between 0.84 and 0.86 (Selvachandran *et al*, 2002). However, the discriminatory power results for LC compare favourably with those models for breast cancer (0.58–0.68) and melanoma (0.62) (Rockhill *et al*, 2003; Cho *et al*, 2005; Tice *et al*, 2005). Future improvements in the discriminatory ability of LC risk models may be possible by the incorporation of biomarkers related to LC risk, top hits from genome-wide association studies, rare variants, or a combination of these with lifestyle and environmental risk factors. Improved LC risk models offer an enormous potential benefit to guide the physician's and the patient's perception of individual risk of disease.

## Figures and Tables

**Table 1:** Lifestyle variables used in the Bach, LLP, and Spitz models

**Variables**	**Bach**	**LLP**	**Spitz**
Cigarettes smoked per day	Yes	No	No
Smoking duration	Yes	Yes	No
Pack-years	No	No	Yes
Cessation duration	Yes	No	No
Age stopped smoking	No	No	Yes
Age	Yes	Used for LC incidence rate	Used for LC incidence rate and LC-free mortality rate
Sex	Yes	Used for LC incidence rate	Used for LC-specific incidence rate and LC-free mortality rate
Family history	No	Yes	Yes
Asbestos exposure	Yes	Yes	Yes
Wood dust exposure	No	No	Yes
Emphysema	No	No	Yes
Hay fever	No	No	Yes
Pneumonia	No	Yes	No
Malignant tumour	No	Yes	No
LC incidence rate	Yes (1-year recursed 5 times)	Yes (modelled for five years)	Yes (SEER rate)
LC-free mortality rate	Yes (1-year recursed 5 times)	No	Yes (NCHS rate)

Abbreviations: LLP=Liverpool Lung Project; LC=lung cancer; SEER=Surveillance and End Results; NCHS=National Center for Health Statistics.

**Table 2:** Demographic characteristics of study population used to compare discriminatory power and accuracy of the Spitz, Bach, and LLP risk models

	**Cases (*N*=1066)**	**Controls (*N*=677)**	***P*-value**
Age (years): mean±s.e.	64.8±9.7	61.1±9.8	<0.001
			
*Smoking status: no. (%)*			
Current	467 (43.8)	175 (25.8)	<0.001
Former	599 (56.2)	502 (74.2)	
			
*Current smokers*			
Smoking duration (years): mean±s.e.	43.9±10.3	38.5±10.5	<0.001
Cigarettes per day: mean±s.e.	29.9±14.8	21.1±11.9	<0.001
Pack-years: mean±s.e.	65.7±37.1	41.2±26.3	<0.001
Family history of cancer (no. % of family members)			
<2	320 (68.5)	130 (74.3)	0.176
⩾2	147 (31.5)	45 (25.7)	
Family history of a smoking-related cancer (no. % of family members)			
0	325 (69.6)	136 (77.7)	0.049
⩾1	142 (30.4)	39 (22.3)	
			
*Former smokers*			
Smoking duration (years): mean±s.e.	34.8±12.3	24.2±12.8	<0.001
Cigarettes per day: mean±s.e.	30.6±16.4	22.9±16.0	<0.001
Pack-years: mean±s.e.	55.3±36.3	30.6±28.5	<0.001
Years since smoking cessation: mean±s.e.	14.1±10.9	19.8±12.1	<0.001
Family history of cancer (no. % of family members)			
<2	393 (65.6)	362 (72.1)	0.023
⩾2	206 (34.4)	140 (27.9)	
Family history of a smoking-related cancer (no. % of family members)			
0	416 (69.4)	387 (77.1)	0.005
⩾1	183 (30.6)	115 (22.9)	

Abbreviation: LLP=Liverpool Lung Project.

**Table 3:** Discriminatory power for the Spitz, Bach, and LLP risk models, overall and stratified by smoking, age, and sex

					***P*-value**	
		**Model**	**Area under the curve**	**Asymptotic 95% confidence interval**	**(1)**	**(2)**
Overall		Spitz	0.69	0.66–0.71		
		Bach	0.66	0.64–0.69	0.07	
		LLP	0.69	0.67–0.71	0.61	0.02
Current smokers		Spitz	0.68	0.64–0.72		
		Bach	0.65	0.60–0.69	0.02	
		LLP	0.66	0.62–0.70	0.23	0.45
Former smokers		Spitz	0.70	0.67–0.73		
		Bach	0.65	0.62–0.68	**0.01**	
		LLP	0.70	0.67–0.73	0.71	**<0.01**
Age group (years)	Sex					
<50	Women	Spitz	0.59	0.46–0.69		
		Bach	0.62	0.49–0.72	0.63	
		LLP	0.62	0.49–0.72	0.64	0.99
<50	Men	Spitz	0.70	0.58–0.78		
		Bach	0.68	0.56–0.77	0.66	
		LLP	0.67	0.56–0.76	0.65	0.96
⩾50	Women	Spitz	0.70	0.66–0.73		
		Bach	0.65	0.61–0.69	0.05	
		LLP	0.69	0.66–0.73	0.86	0.04
⩾50	Men	Spitz	0.68	0.64–0.71		
		Bach	0.67	0.63–0.70	0.63	
		LLP	0.70	0.67–0.74	0.13	0.09

Abbreviation: LLP=Liverpool Lung Project.

(1) *P*-value comparisons for the Spitz and Bach models and the Spitz and LLP models.

(2) *P*-value comparisons for the Bach and LLP models.

Bold values represent results still significant to the 5% level after using the Bonferroni method for multiple corrections.

**Table 4:** Positive predictive and negative predictive values (PPV and NPV, respectively) examination using a predictive cutoff of 2.5%

			***P*-value**			***P*-value**	
**Smoking status**	**Model**	**PPV**	**(1)**	**(2)**	**NPV**	**(1)**	**(2)**
All	Spitz	0.882			0.450		
	Bach	0.809	0.009		0.447	0.911	
	LLP	0.759	<0.001	0.049	0.560	**<0.001**	**<0.001**
Former	Spitz	0.855			0.520		
	Bach	0.836	0.851		0.526	0.832	
	LLP	0.726	<0.001	0.058	0.649	**<0.001**	**<0.001**
Current	Spitz	0.919			0.323		
	Bach	0.804	0.002		0.354	0.418	
	LLP	0.809	<0.001	0.943	0.384	0.110	0.507

Abbreviation: LLP=Liverpool Lung Project.

(1) Comparison of the Spitz and Bach and the Spitz and LLP models.

(2) Comparison of Bach and LLP values.

Bold values represent results still significant to the 5% level after using the Bonferroni method for multiple corrections.

**Table 5:** Clinical utility of the Spitz, Bach, and LLP risk models estimated as percentage of participants with risk estimates >2.5, 5.0, and 7.5%

**Model**	**Risk⩾2.5%**	**Risk⩾5.0%**	**Risk⩾7.5%**
*Percentage (%) of cases*			
Spitz	26.6	6.8	2.2
Bach	30.2	15.5	6.4
LLP	66.7	45.5	31.2
			
*Percentage (%) of controls*			
Spitz	5.6	0.7	0.0
Bach	11.2	2.4	1.2
LLP	33.4	15.1	7.7

Abbreviation: LLP=Liverpool Lung Project.

**Table A1:** Estimated *α*-values for determination of 5-year absolute risk for the LLP risk model (Cassidy *et al*, 2008; SEER, 2005)

	**Male**		**Female**	
**Age group (years)**	**Incidence rate^a^**	***α*-value**	**Incidence rate^a^**	***α*-value**
40–44	10.78	−9.42	11.03	−9.29
45–49	25.49	−8.56	23.19	−8.54
50–54	56.60	−7.76	45.51	−7.86
55–59	116.58	−7.02	93.93	−7.13
60–64	221.18	−6.37	164.90	−6.56
65–69	346.77	−5.91	246.85	−6.15
70–74	478.10	−5.57	318.69	−5.88
75–79	564.36	−5.37	344.67	−5.79
80–84	532.36	−5.43	308.28	−5.91

Abbreviation: SEER=Surveillance and End Results. ^a^Lung cancer incidence rate per 100 000 person-years. SEER.

**Table A2:** Lung cancer and mortality rates per 100 000 (excluding lung cancer) by age and sex (Whites only) (National Center for Health Statistics, 2003)

	**Men**		**Women**	
**Age (years)**	**Incidence**	**Mortality**	**Incidence**	**Mortality**
20–24	0.26	129.10	0.36	46.70
25–29	0.51	120.00	0.62	50.00
30–34	0.99	136.40	1.26	64.60
35–39	3.40	185.30	4.16	99.80
40–44	10.78	275.10	11.03	153.20
45–49	25.49	400.70	23.19	218.80
50–54	56.60	560.00	45.51	313.40
55–59	116.58	786.90	93.93	479.10
60–64	221.18	1210.20	164.90	762.90
65–69	346.77	1855.10	246.85	1197.00
70–74	478.10	2947.40	318.69	1968.30
75–79	564.36	4836.40	344.67	3306.10
80–84	532.36	7980.70	308.28	5761.20
>85	498.44	15 559.40	266.72	14 016.20

## Appendix

See Table A1 and Table A2
